# Implementation of Machine Learning in Flat Die Extrusion of Polymers

**DOI:** 10.3390/molecules30091879

**Published:** 2025-04-23

**Authors:** Nickolas D. Polychronopoulos, Ioannis Sarris, John Vlachopoulos

**Affiliations:** 1 Department of Mechanical Engineering, University of West Attica, Ancient Olive Grove Campus, Egaleo, 12241 Athens, Greece; sarris@uniwa.gr; 2 Department of Chemical Engineering, McMaster University, Hamilton, ON L8S 4L7, Canada

**Keywords:** dataset generation, polymer rheology, ML algorithms, SHAP analysis

## Abstract

Achieving a uniform thickness and defect-free production in the flat die extrusion of polymer sheets and films is a major challenge. Dies are designed for one extrusion scenario, for a polymer grade with specified rheological behavior, and for a given throughput rate. The extrusion of different polymer grades and at different flow rates requires trial-and-error procedures. This study investigated the application of machine learning (ML) to provide guidance for the extrusion of sheets and films with a reduced thickness, non-uniformities, and without defects. A dataset of 200 cases was generated using computer simulation software for flat die extrusion. The dataset encompassed variations in die geometry by varying the gap under a restrictor, polymer rheological and thermophysical properties, and processing conditions, including throughput rate and temperatures. The dataset was used to train and evaluate the following three powerful machine learning (ML) algorithms: Random Forest (RF), XGBoost, and Support Vector Regression (SVR). The ML models were trained to predict thickness variations, pressure drops, and the lowest wall shear rate (targets). Using the SHapley Additive exPlanations (SHAP) analysis provided valuable insights into the influence of input features, highlighting the critical roles of polymer rheology, throughput rate, and the gap beneath the restrictor in determining targets. This ML-based methodology has the potential to reduce or even eliminate the use of trial and error procedures.

## 1. Introduction

Flat dies are used in the production of thermoplastic sheets, films, oriented films, and extrusion coatings. A molten polymer is supplied from an extruder through a melt tube to a die, which terminates at a wide rectangular cross-section with a small gap. For thicknesses less than 0.25 mm, the process is referred to as-cast film extrusion, and for the sheet extrusion of thicker products, after exiting the flat die, the extrudate is cooled on chilled rollers and solidifies. The cooling rate determines several important properties of the finished product. Some films are stretched by a tenter frame, as in the case of biaxially oriented polypropylene (BOPP). Films are used in food packaging, protective wraps, and medical and industrial applications. Sheets are frequently shaped by thermoforming for refrigerator liners, containers, yogurt and drink cups, glass replacements, back lighting display panels, and other products.

**Figure 1:**
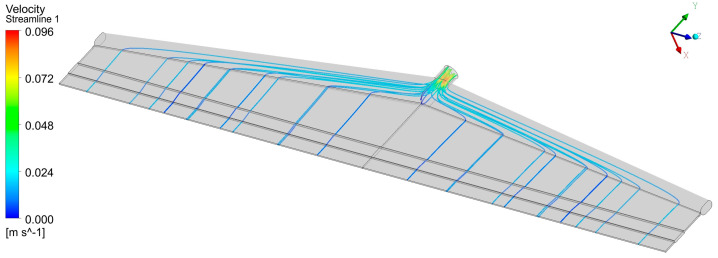
Stream-tracers for a typical “coat-hanger” flat die, obtained from a fully 3D numerical simulation. Computational mesh size: 1.3 million cells, after a mesh convergence study.

**Figure 2:**
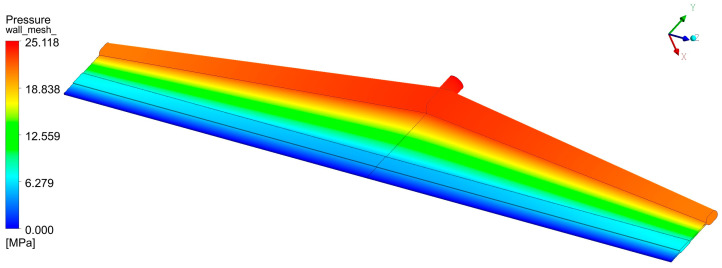
Pressure distribution for the die of Figure 1.

The most important function of the die is to spread the molten polymer as uniformly as possible into the rectangular shaping die lips so that a wide sheet or film of uniform thickness is produced [1]. Thickness uniformity in both the machine and transverse directions is crucial. Machine direction uniformity can be improved by the addition of a gear pump between the extruder and the die to provide a steady melt flow. Transverse direction uniformity is a very challenging problem, especially due to die widths, which have increased dramatically in recent years, frequently reaching 4–5 m and occasionally exceeding 10 m. Obviously, the polymer melt would follow the path of least resistance flowing towards the die center, thereby producing a thicker sheet in the central area, being thinner near the edges. The flow channels should be designed in such a way as to exhibit more resistance in the central area and less resistance near the edges. Years ago, T-slot dies were used with a distribution channel perpendicular to the supply tube. Nowadays, the ‘coat-hanger’ geometry is by far the most common. Figure 1 shows the stream-tracers in a coat-hanger die 1 m wide, and Figure 2 shows the pressure field obtained by a fully 3D flow simulation using the Ansys Fluent package (Ansys Fluent Release 2022 R1) [2]. Pressures from 10 MPa to 40 MPa are common in such dies. It can easily be calculated that very large forces are developed due to the large surface area of flat dies used in industrial production installations. Flat dies are clamped together at the edges, and deflection may occur, with the largest magnitude at the center. This is usually referred to as clamshelling. It results in an increased flow in the central region, which must be compensated for through lip adjustments.

For polymer melt flow simulation, the viscosity as a function of the shear rate must be known, and it is usually expressed either by the power-law, Carreau–Yasuda, or Cross models.

(1) $$\eta \left(\dot{\gamma },T\right)=m\left(T\right){\dot{\gamma }}^{n-1},$$

(2) $$\eta \left(\dot{\gamma },T\right)={\;\eta }_{0}\left(T\right){\left[1+{\left(\lambda \dot{\gamma }\right)}^{\alpha }\right]}^{\frac{n-1}{a}},$$

(3) $$\eta \left(\dot{\gamma ,}T\right)=\frac{\eta_{0}\left(T\right)}{1+{\left(\lambda \dot{\gamma }\right)}^{1-n}},$$

where m is the consistency index (the viscosity value at $\dot{\gamma }$ = 1/s), n is the power-law exponent, $\eta_{0}$ is the zero shear viscosity, and a and λ are fitting parameters. Both m and $\eta_{0}$ are functions of temperature T. Temperature dependence is usually expressed by the Arrhenius or simple exponential models.

(4) $$\begin{array}{c} m\left(T\right)\\ or\\ \eta_{0}\left(T\right) \end{array}=\left\{\begin{array}{c} m_{ref}\\ or\\ \eta_{0,ref} \end{array}\right\}\cdot exp\left[\frac{E_{a}}{R}\left(\frac{1}{T}-\frac{1}{T_{ref}}\right)\right],$$

(5) $$\begin{array}{c} m\left(T\right)\\ or\\ \eta_{0}\left(T\right) \end{array}=\left\{\begin{array}{c} m_{ref}\\ or\\ \eta_{0,ref} \end{array}\right\}\cdot exp\left[-b\left(T-T_{ref}\right)\right],$$

where $m_{ref}$ and $\eta_{0,ref}$ are measured at a refence temperature $T_{ref}$, $E_{a}$ is the activation energy, and R is the gas constant. While viscoelastic constitute equations are used for some simple problems like extrudate swell and entry flow in a contraction, they are not used in routine industrial die design due to challenges related to numerical convergence and the extraordinarily long computer times required. Thermal conductivity k and specific heat $C_{p}$ are also important, but they do not vary much within the temperature range of melt extrusion. Melt density ρ is usually about 15% less than the solid density of common polymers and can easily be measured [3].

The conservation of mass momentum and energy equations are as follows [3,4,5]:

(6) ∇·v=0,

(7) −∇p+∇·τ=0,

(8) $$\rho C_{p}v\cdot \nabla T=k\nabla^{2}T+\tau :\nabla v,$$

where v is the velocity vector, p is the pressure, τ is the stress tensor, k is thermal conductivity, and the term τ:∇v represents the viscous dissipation (frictional heating). The stress tensor is usually written in terms of the Generalized Newtonian Fluid (GNF) model in the following form:

(9) $$\tau =\eta \left({ΙΙ}_{D}\right)2D,$$

where $\eta \left({ΙΙ}_{D}\right)$ is the viscosity and ${ΙΙ}_{D}$ is the second invariant of the strain rate tensor D, given by the following:

(10) $$D=\frac{1}{2}\left[\nabla v+{\nabla v}^{T}\right],$$

These equations are usually solved by finite element [6] or finite volume [7] methods, and they provide detailed information on the flow field. In flat die extrusion, the main objective is to design a die with minimal transverse direction non-uniformity. Using computer flow simulation software, an iterative design process can be employed on the computer screen. In the first step, the designer must assume the initial geometrical dimensions which are likely to produce a sheet or film with the desired thickness and width. Several iterations are necessary to achieve a geometry capable of producing an acceptable thickness variation, which should not be more than perhaps 5%. Even a person experienced in the design of such dies is likely to require three dozen trial-and-error attempts on the computer screen for a new die for a new material. Fully 3D simulations are very time-consuming, and for this reason, the momentum and continuity equations shown above are frequently simplified to the generalized Hele-Shaw approximation with the assumption of narrow-gap geometry [1,8].

(11) $$\frac{\partial }{\partial x}\left[S\left(x,y\right)\frac{\partial p}{\partial x}\right]+\frac{\partial }{\partial y}\left[S\left(x,y\right)\frac{\partial p}{\partial y}\right]=0,$$

where the quantity $S\left(x,y\right)$ is referred to as flow conductance and is defined as follows:

(12) $$S\left(x,y\right)=\int_{0}^{h}\frac{z^{2}dz}{\eta \left(x,y,z\right)},$$

where h is the gap in the z-direction. The primary variable is the pressure p and after finding it, the gap-wise average velocity components can be calculated from the following:

(13) $$\bar{V_{x}}=-\frac{S}{h}\frac{\partial p}{\partial x}\;\;\;\;\;\;\;\;\;\;\bar{V_{y}}=-\frac{S}{h}\frac{\partial p}{\partial y},$$

and the full velocity distributions are given by the following:

(14) $$V_{x}\left(z\right)=-\frac{\partial p}{\partial x}\int_{0}^{h}\frac{z^{\prime }dz^{\prime }}{\eta \left(z^{\prime }\right)}\;\;\;\;\;\;\;\;\;V_{y}\left(z\right)=-\frac{\partial p}{\partial y}\int_{0}^{h}\frac{z^{\prime }dz^{\prime }}{\eta \left(z^{\prime }\right)},$$

where z′ is a dummy variable of integration. The optimization of flat die design has been attempted by some investigators [9,10,11,12], but this methodology has not been applied beyond publications in scholarly journals. Flat dies are currently designed by using iterative simulation procedures, as explained above.

A reduction in thickness non-uniformity can be further achieved by equipping the die with a restrictor bar. The restrictor is used to increase resistance to flow near the center or wherever else it might be required. Most flat dies include lip gap adjustment systems for fine-tuning the uniformity. They can be simple manually operated adjusting bolts or automated systems. Automated systems involve downstream web thickness measurement and feedback control.

The maximum possible line speed (throughput rate) is usually determined by the shear stress at the die lip wall. When a critical value is exceeded (0.14 MPa for HDPE), polyolefins (with the exemption of LDPE) and most other common polymers exhibit a surface irregularity known as sharkskin [13,14,15,16], which is visible to the naked eye and usually appears as ridges perpendicular to the flow direction. Polymer processing aids (PPAs) are used to increase the output rate for sharkskin-free surface, but most of them contain Per- and Poly-fluorinated substances (PFASs), which have recently been found to pose health risks [17,18,19]. However, several corporations are actively involved in developing PFAS-free alternatives. PPAs promote wall slip, thereby ‘postponing’ the sharkskin effect to higher output rates.

For extrudate sharkskin, which appears at high lip wall shear rates and stresses, there have been hundreds of publications. However, there are hardly any publications on extrusion defects occurring at low wall shear rates and affecting transparent films produced by flat film or blown film extrusion [1], which are known to extrusion operators and machine manufacturers. It has been observed in transparent and semi-transparent film extrusion that when regions of the die have a wall shear rate lower than a certain value, the optical clarity is inferior. For HDPE, when the wall shear rate is very low, the slow-moving melt layer near the wall tends to cross-link due to a high temperature. Cross-linking impairs film clarity. Other polymers may experience other forms of degradation. The deterioration of optical properties may be immediately visible after extrusion begins, but frequently, the die may operate for some time before defects are noticeable. Flat die manufacturers usually design dies for a lowest wall shear rate of 10 s^−1^. For some temperature-sensitive materials, the lowest acceptable shear rate is higher. Of course, the wall shear rate might be increased by increasing the output rate. However, such a remedy is not always possible due to limitations upstream (extruder capacity) or downstream (cooling rate).

Flat dies designed with the use of a suitable software package can meet the requirements for high-quality sheets and films. However, die flow channels are designed for one processing scenario, involving a specific material for the desired throughput rate. Of course, a die is expected to be used for different polymers with different properties, and the desired throughput rates may vary substantially. The objective of the present investigation is to develop a methodology, based on Artificial Intelligence (AI) and machine learning (ML), which can be used in providing guidance for the production of sheets or films with as uniform a thickness as possible and free from any extrusion defects, for a wide range of materials and throughput rates.

Artificial Intelligence (AI) techniques have exerted a significant influence on various technological areas. Machine learning (ML) has recently emerged as an AI branch focusing on the use of data and algorithms to identify patterns, make predictions, and progressively improve prediction accuracy with minimal, if any, human intervention. Supervised learning is a common machine learning process that typically involves learning a mapping between given input and output pairs. The success of ML and AI methodologies in several technological areas is due to the availability of extremely large digital datasets. For instance, ML for pattern identification in computer vision has been successful because of the existence and immediate availability of millions of images. However, datasets for the design of process equipment and machine structures are impossible or difficult to find. Yet, there are some exceptions. For airfoil design, the UIIC Airfoil Database [20] is a dataset of 1600 real designs of airfoils, recently updated with lift and drag values. Moreover, Wollstadt et al. [21] produced a dataset of more than 10,000 different vehicle hood shapes, along with mechanical performance data, and used machine learning to predict the hood mechanical performance and build metamodels for design optimization. Regenwetter et al. [22] used ML for the prediction of the structural performance of bicycle frames. Their dataset was constructed from various bicycle frames sourced from designers who used a software package.

For the present investigation, ideally, a dataset could have been collected from an extrusion plant where a die was used for polymers with different rheological and thermophysical properties and in different operating conditions. Such data are available to corporations that have been producing flat sheets or films for many years, but nothing similar is available in the open literature. Consequently, the present team of authors decided to generate a dataset using computer simulation.

## 2. Results and Discussion

Three powerful machine learning (ML) models (algorithms) were assessed in the present investigation by plotting the predicted values against those obtained from numerical simulations. Data points on the diagonal (y=x) indicate a zero-error prediction. The data scatter around the diagonal provides a quick visual assessment of the ML model for a training dataset and a testing dataset.

**Figure 3:**
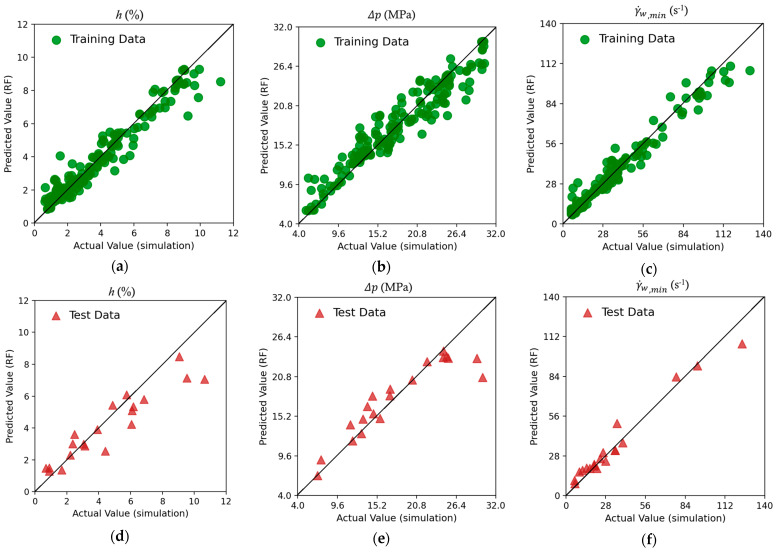
Scatter plots of the three targets using Random Forest (RF). Training dataset for (**a**) thickness variation h; (**b**) pressure drop Δp; and (**c**) lowest wall shear rate ${\dot{\gamma }}_{w,min}$ and testing dataset for (**d**) thickness variation; (**e**) pressure drop Δp; and (**f**) lowest wall shear rate ${\dot{\gamma }}_{w,min}$.

Figure 3 shows the scatter plot of the predictions by the Random Forest (RF) model versus the numerical simulations. Figure 3a–c show the scatter plots for the training dataset. The data points are concentrated around the diagonal line, indicating that the model exhibits a satisfactory degree of precision and relatively small deviations for the training data. This suggests that the RF model has learned the underlying patterns of the dataset, demonstrating a satisfactory predictive capability. The prediction of the thickness variation target h and the pressure drop target Δp appear to be somewhat more scattered than that of the lowest shear rate ${\dot{\gamma }}_{w,min}$, The scatterplots for the test dataset (Figure 3d–f) provide insight into the capability of the RF model to predict targets not included in the training. The alignment of data points with the diagonal line appears to follow the trend of the training dataset, but for the h and Δp targets, the majority of the points appear to be well off the diagonal line, especially for higher values. However, most of the test data for the lowest wall shear rate target are more clustered around the diagonal line, which suggests that RF likely generalizes better for this target. The overall performance suggests that RF regression remains a satisfactory technique for predicting these complex targets, with a reasonable generalization capability.

**Figure 4:**
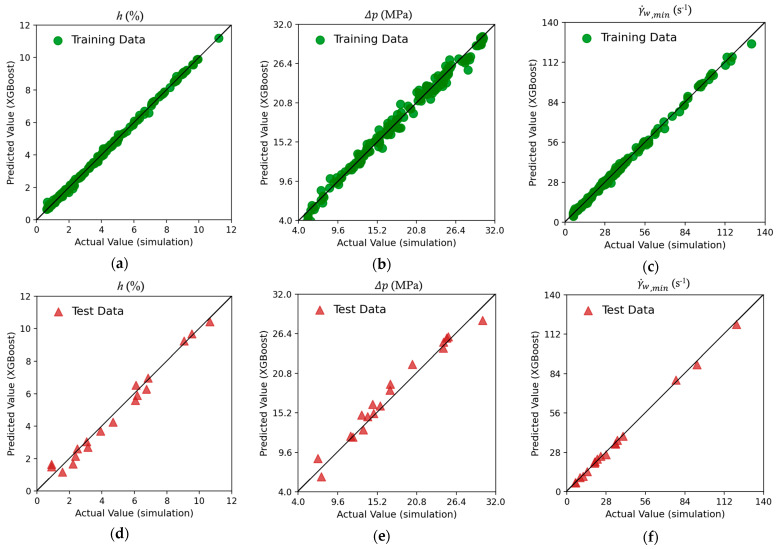
Scatter plots of the three targets using XGBoost. Training dataset for (**a**) thickness variation h; (**b**) pressure drop Δp; and (**c**) lowest wall shear rate ${\dot{\gamma }}_{w,min}$ and testing dataset for (**d**) thickness variation h; (**e**) pressure drop Δp; and (**f**) lowest wall shear rate ${\dot{\gamma }}_{w,min}$.

Figure 4 shows the scatter plots using the XGBoost ML algorithm. In the training data (top row, Figure 4a–c), it is observed that the predictions for the h(%) and ${\dot{\gamma }}_{w,min}$ targets closely follow the diagonal line, indicating that the XGBoost regressor is able to learn and predict the training dataset with a high accuracy. A somewhat lower accuracy is observed for the Δp target, as shown in Figure 4b. The data spread around the diagonal for all targets exhibits much less deviation than the RF model in Figure 3. This behavior suggests that the XGBoost model has effectively captured the underlying patterns in the training data. For the testing dataset, Figure 4d–f, the data are gathered closely around the diagonal line and follow the scatter pattern of the training dataset well, suggesting that overfitting, if any, is minimal. Therefore, the XGBoost model performance indicates that the model has successfully captured the key relationships in the data and makes reasonable generalizations. XGBoost, in handling numerical regression tasks, manages to produce high-fidelity approximations of complex relationships while maintaining a relatively low prediction error.

**Figure 5:**
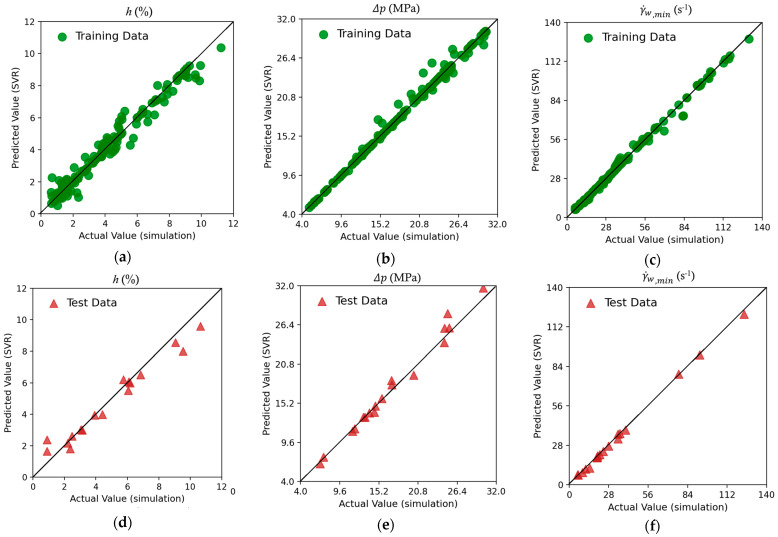
Scatter plots of the three targets using Support Vector Regressor (SVR). Training dataset for (**a**) thickness variation h; (**b**) pressure drop Δp; and (**c**) lowest wall shear rate ${\dot{\gamma }}_{w,min}$ and testing dataset for (**d**) thickness variation h; (**e**) pressure drop Δp; and (**f**) lowest wall shear rate ${\dot{\gamma }}_{w,min}$.

Figure 5a–c show the results for the training dataset using the Support Vector Regressor (SVR). Immediate comparison can be performed on the basis of the predictions made by XGboost in Figure 4 above. The SVR model demonstrates a robust predictive performance on the training dataset, as evidenced by the close alignment of the predicted values with the diagonal line. However, in comparison to the XGBoost regressor, an increased dispersion of data is observed, particularly with the h(%) target (Figure 5a) and, to some extent, with the lowest shear rate at the wall target (Figure 5c). For the case of the pressure drop target (Figure 5b), SVR makes very good predictions at low pressure drop values, but a high data spread is observed at higher pressure drop values. The test data results reveal notable differences between the XGBoost and SVR models. The thickness variation predictions for SVR in Figure 5d exhibit more deviation from the diagonal compared to XGBoost. For the Δp target in Figure 5e and ${\dot{\gamma }}_{w,min}$ target in Figure 5f, SVR’s test predictions remain fairly close to the diagonal, though they display slightly larger deviations than XGBoost’s results.

Table 1 presents the predictive performances of the three ML algorithms in terms of the $R^{2}$ value, RMSE, and MAE. The RF algorithm generally exhibits the lowest $R^{2}$ values and highest error metrics for all three targets, in accordance with the observations obtained from the scatter plots. For the thickness variation and lowest shear rate at the wall targets, XGBoost exhibits the highest $R^{2}$ values, 0.954 and 0.985, respectively and, at the same time, has the lowest errors for these two targets, namely RMSE = 0.591 and RMSE = 1.619, respectively. The SVR model exhibits lower $R^{2}$ values and higher error metrics for these two targets. For the case of the pressure drop target, SVR shows a slightly higher $R^{2}$ value and slightly lower errors than XGBoost. Overall, the performance of the XGBoost model was deemed better than that of the SVR. Therefore, we carry out further analysis with the XGBoost algorithm.

**Figure 6:**
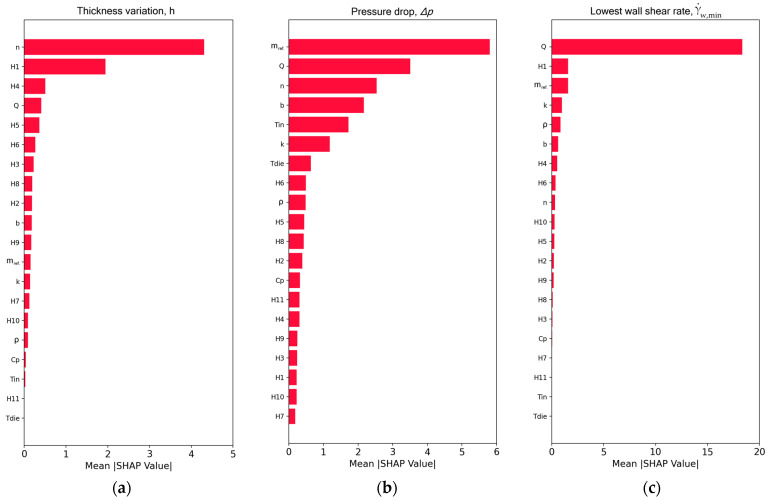
SHAP feature importance plot for the XGBoost model. (**a**) Thickness variation, h; (**b**) pressure drop (ΔP); and (**c**) lowest wall shear rate, ${\dot{\gamma }}_{w,min}$.

The predictions obtained from the XGBoost model are further interpreted by SHAP analysis in two different ways. First, the feature importance of the input variables is examined, which expresses their impact on the predictions made by the XGBoost model. This information is given in Figure 6 for the three targets. In all cases, the horizontal axis corresponds to the average of the absolute SHAP values of the dataset and the vertical axis corresponds to all the features. It can be seen from Figure 6a that the power-law index n exhibits the highest influence on the predictions of the thickness variation. This aligns very well with observations from experiments on several polymers that shear-thinning has a very high impact on lateral thickness distribution. The second most influential feature is the gap under the restrictor bar (H1) located on the axis of symmetry of the flat die analyzed. At this location, the gap was varied a lot in the numerical simulations for the purpose of obtaining a low transverse thickness variation. Yet, the feature importance ranking suggests that this parameter is less important than shear-thinning. The gaps at the other locations of the restrictor bar and the polymer thermophysical properties have a rather small effect on the XGBoost predictions. For the pressure drop target in Figure 6b, the most influential feature in the XGBoost prediction is the consistency index, $m_{ref}$, followed by the throughput rate Q, the power-law index n, the temperature sensitivity b, the melt entering temperature $T_{in}$, and the polymer thermal conductivity k. The rest of the features contribute less to the XGBoost predictions. It is noteworthy that, while for thickness variation, the algorithm relied mostly on two features, we see that for pressure drop, there were more contributions. From the physical perspective, the flow in the present die can be roughly approximated as the flow of a shear-thinning fluid between two flat plates, where, according to textbooks [3,4,5], $\Delta p\sim mQ^{n}$. The pressure drop is linearly correlated with m, but is less dependent on Q, since 0<n<1, fully in agreement with the SHAP analysis. Also, since m=m(T), the b, $T_{in}$, and k features have a high importance in the decisions made by XGBoost. Finally, for the lowest wall shear rate in Figure 6c, XGBoost relied mainly on one feature, the flow rate Q, with H1 and $m_{ref}$ having significantly less impact.

**Figure 7:**
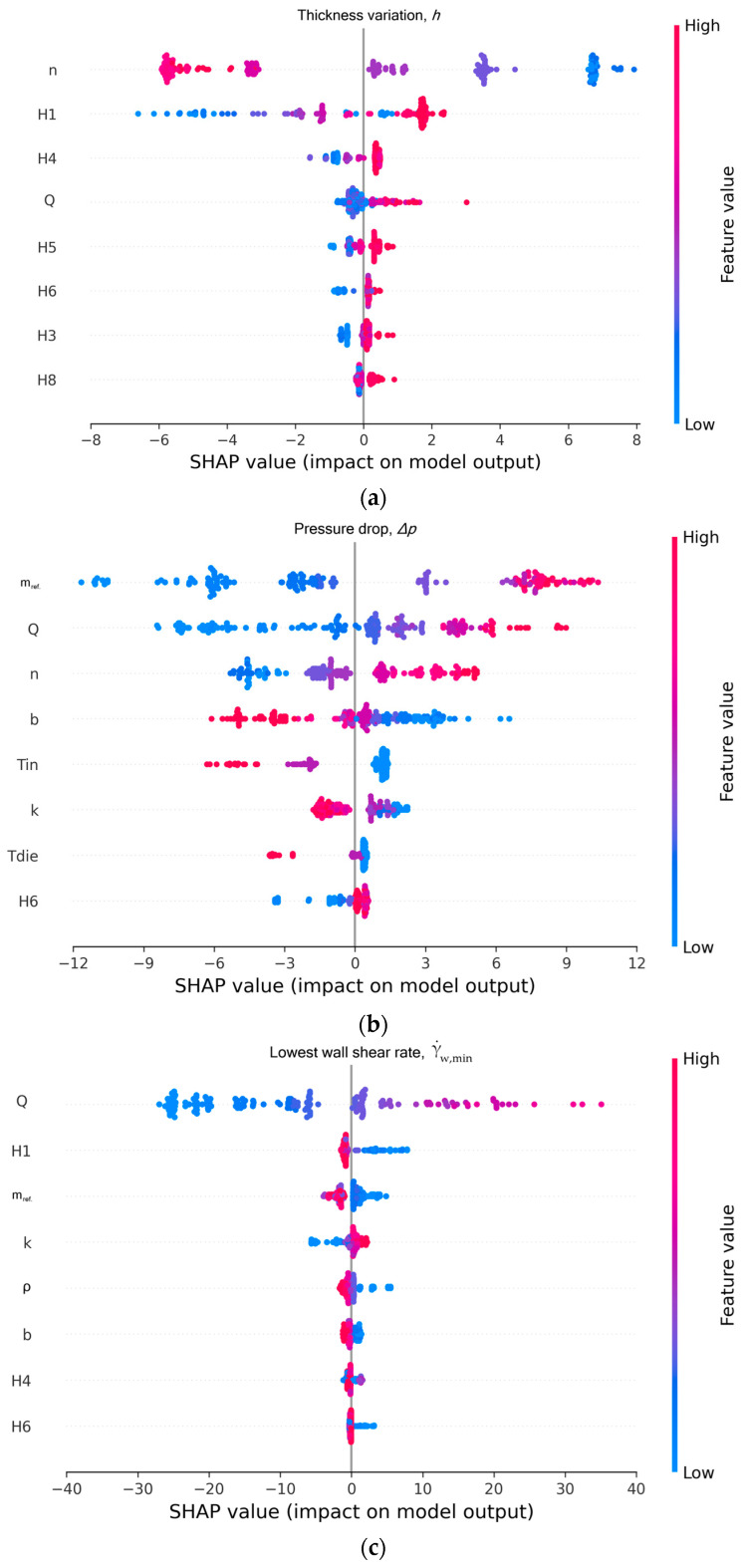
SHAP summary plot for XGBoost. (**a**) Thickness variation, (h); (**b**) pressure drop (ΔP); and (**c**) lowest wall shear rate, ${\dot{\gamma }}_{w,min}$.

More information on the decisions made by XGBoost can be obtained from the plot shown in Figure 7, frequently referred to in the ML literature as a SHAP summary plot. It shows the distribution of SHAP values for each feature, elucidating the directional feature influence on each target. In this figure, the *x*-axis corresponds to the SHAP values and the *y*-axis lists the features in descending order of importance. Note that, for visualization purposes, we show only the top eight contributing features for each target. Each point in the plot corresponds to an individual sample within the dataset. The color encoding to the right of each plot represents the feature magnitude. The blue color is for low feature values and the red color is for high feature values. The horizontal placement of each point signifies the impact of the feature on the model’s prediction values. It simply indicates whether a lower or higher feature value leads to a lower prediction value (negative SHAP value) or a higher prediction value (positive SHAP value). For example, for the h(%) target (Figure 7a), increasing the power-law index n leads to predictions of lower thickness variation values, since the corresponding SHAP values are negative. In other words, higher n values lead to an extruded sheet with lower thickness variation. A higher power-law index suggests that the polymer is less shear-thinning, thereby producing more uniform films or sheets. Is it worth mentioning that reducing the gap in the H1 bar restrictor location also leads to a decreased thickness variation. The same behavior is also noticed for the H4 location, but with a far lower contribution. For the pressure drop target Δp (Figure 7b), increasing the consistency index $m_{ref}$, the throughput rate Q, and the power-law index n leads to an increase in Δp, which is reasonable from the physical perspective. On the other hand, increasing the temperature sensitivity b and the melt entering temperature $T_{in}$ decreases the pressure drop. This is also reasonable, because increasing these two features leads to decrease in viscosity, which finally leads to pressure drop decrease. Finally, it is shown in Figure 7c that increasing the throughput rate leads to an increase in the lowest shear rate at the wall, which is obvious, and the not so apparent impacts of other features.

## 3. Materials and Methods

### 3.1. Dataset Generation for a Base Case Flat Extrusion Die

As explained in the introduction, the objective is to employ ML for the successful operation of extrusion through a flat die of different polymers and at different throughputs, which means a reduction in the transverse thickness variation, the maintenance of pressure at an acceptable level, and an increase in the lowest wall shear rate above a certain value. The shear rate and the corresponding shear stress at the die lip wall are also important quantities related to the onset of sharkskin, but they can easily be calculated from simple equations of flow between two parallel plates [3,4,5]. The dataset was generated using a computer simulation. There are two options for computer simulation. Either a fully 3D flow analysis or a 2D one using the Hele-Shaw flow approximation. While 3D flow analysis is straightforward for a specific flat die geometry and a specific material rheology, as shown in Figure 1 and Figure 2, it is extremely challenging to produce a large dataset (200 cases in the present project) due to numerical convergence issues associated with geometrical complexities and the shear rate and temperature dependence of viscosity. A numerical grid might be suitable for one set of rheological parameters, but not for the next. Also, the required computation times are very large. It was, therefore, decided to use a software package called Flatcad (Flatcad v.3.0) [23,24], which is essentially a solution to the Hele-Shaw equation—Equation (10)—and also includes the temperature dependence of viscosity, using the control volume method. There are several dies operating in industry today which have been designed using Flatcad.

**Figure 8:**
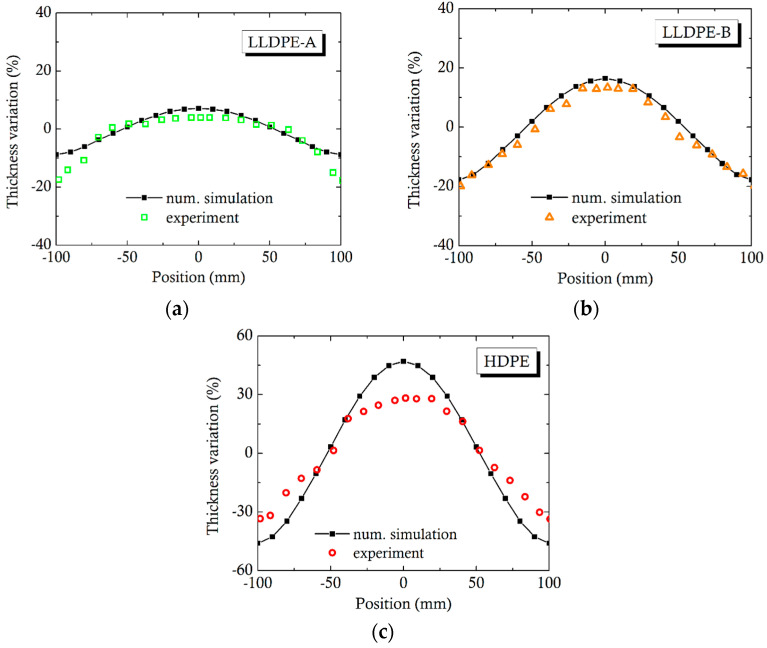
Comparison of Flatcad software [23] to experiments by Vlcek et al. [26] for three different polyethylene types: (**a**) LLDPE grade A; (**b**) LLDPE grade B, and (**c**) HDPE.

Flatcad divides the domain of integration into control volumes with pressure into the main (x) and the transverse $\left(y\right)$ flow directions, maintaining mass, momentum, and energy balance [24]. To some extent, this method is similar to that of Köpplmayr and Mietlinger [25]. The current version of Flatcad was tested against some available experimental data [26] for a small die with a 200 mm width, carried out in the laboratory of the third author of the present publication. A satisfactory comparison of the thickness non-uniformity of the sheet produced is shown in Figure 8 for three materials, LLDPE-A, LLDPE-B, and HDPE, with viscosity and temperature sensitivities shown in Table 2. The differences between the simulation and experiment for HDPE were mostly due to experimental challenges in conducting local thickness measurements on frozen extruded tape, rather than in the melt assumed in the computer software. HDPE was more crystalline than the two LLDPEs and exhibited more shrinkage, and, consequently, more difficulty in measuring local thickness.

**Figure 9:**
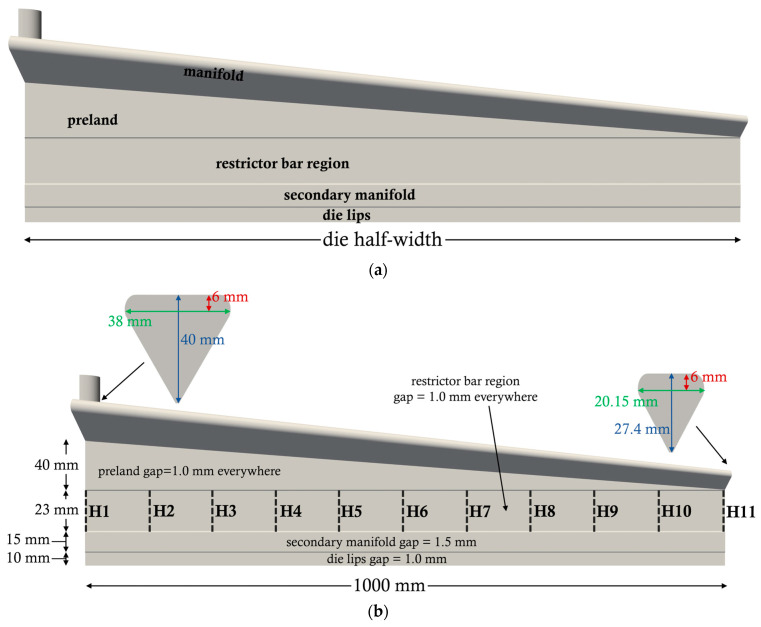
Schematic of the simulated flat extrusion die with deactivated restrictor bar. (**a**) Notation of the die regions and (**b**) die dimensions.

To generate the dataset for ML, the dimensions of an industrial production die were slightly modified. The width of the die lips was 2000 mm. As shown in Figure 9a, the die included a manifold, pre-land (land denotes parallel walls), a restrictor bar, a secondary manifold, and a die lip section (also called land). The die dimensions are shown in Figure 9b. H1, H2, and H3 to H11 indicate the sides of the 10 control volumes in the transverse (y-direction) where the thickness is calculated. There are also 5 rows of 10 control volumes in the flow direction (x-direction). In total, there are 50 control volumes for this (half) die. A total of 200 simulations were carried out and the inputs (features) and outputs (targets) are shown in Table 3. The inputs in this table are based on industrial experience and are in accordance with a previous publication [1] on flat die design by two of the present authors. For example, the power-law exponent n range is between 0.25 for a polymer with a broad molecular weight distribution (MWD) and 0.55 for a narrow MWD. Similarly, the other important material parameters were chosen for commodity polymers used in extrusion for film and sheet production [27]. The gap size range beneath the restrictor was also based on knowledge of industrial extrusion operations.

**Figure 10:**
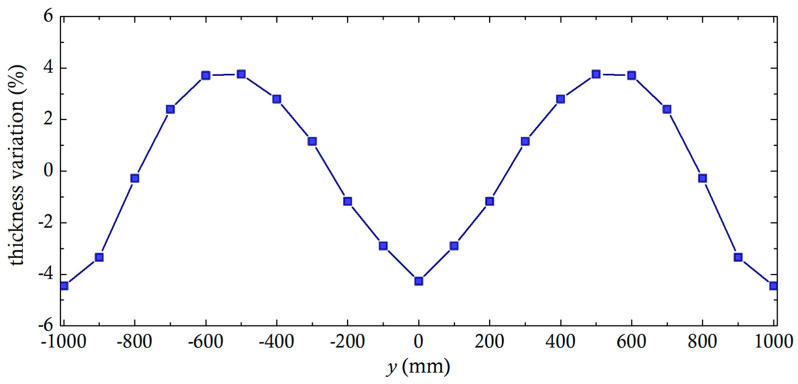
Base case flat die thickness variation obtained via Flatcad [23] with deactivated restrictor bar.

Of course, the most important result is the transverse thickness variation. The original die design, where the restrictor bar is not activated (gap beneath all bar restrictor locations = 1 mm), has a transverse thickness variation shown in Figure 10. The polymer melt has a consistency index of m=10,000 Pa·s^n^ and n = 0.45, a throughput of 400 kg/h, a pressure of 24.98 MPa, and a thickness variation somewhat less than ±5%.

### 3.2. Dataset Initialization and Machine Learning Models

Traditionally, in machine learning (ML), the dataset is split into two subsets of different data proportions. In the primary subset, known as the training dataset, the model has access to both inputs and corresponding outputs. The testing dataset serves as an independent set used to assess the trained model’s performance on unseen data. In total, 90% of the original dataset is allocated for training, while the remaining 10% is designated as the testing dataset. This proportion is frequently used, as explained in previous ML publications [28,29,30].

A key step in ML regression is data pre-processing. A common technique followed is feature (input) scaling. Without scaling, features with larger numerical values may dominate the model’s learning process. This eventually leads to features with smaller ranges to have a reduced impact [31]. Following Karakasidis et al. [32] and Benos et al. [33], features are scaled using the following equation:

(15) $$X_{s}=\frac{X-\bar{X}}{\sigma },$$

where $X_{s}$ denotes the scaled feature, X is the unscaled one, $\bar{X}$ is the mean value of the feature, and σ is the standard deviation.

The following ML algorithms were employed: (i) Random Forest, (ii) eXreme Gradient Boosting (XGBoost), and (iii) Support Vector Regressor (SVR). The architectures of these algorithms are briefly summarized below.

The Random Forest (RF) algorithm is a robust ensemble learning method for both classification and regression analysis. It employs a strategy of constructing an ensemble of decision trees in parallel, each trained independently on a distinct subset of the original dataset [32]. Prediction is subsequently achieved through the aggregation of individual tree outputs, utilizing a majority voting mechanism for classification tasks and an arithmetic mean for regression. For the case of regression, like in the present study, predictions are made by [34], as follows:

(16) $$Y_{pred.}=\frac{1}{N}\sum_{i=1}^{N}Y_{i}\left(X\right)$$

where N is the total number of trees in the forest and $Y_{i}\left(X\right)$ is the prediction made by the i-th decision tree for a given input X.

The eXtreme Gradient Boosting (XGBoost) machine learning algorithm is an ensemble learning method. XGBoost algorithm combines multiple decision trees to build a more accurate and robust model. Unlike RF, where trees are trained independently, XGBoost builds trees sequentially, with each new tree focusing on correcting the errors of the previous ones (boosting) [35,36]. The predictions are made by summing the outputs of the trees by the following equation [37]:

(17) $$Y_{pred.}=\sum_{m=1}^{M}Y_{m}\left(X\right),$$

where M is the total number of trees in the model and $Y_{m}\left(X\right)$ is the prediction from the m-th decision tree for a given input X.

The Support Vector Regressor (SVR) extends the principles of Support Vector Machines (SVMs) to address regression problems [38]. While SVMs are predominantly employed for classification tasks, through the identification of an optimal separating hyperplane in a high-dimensional feature space, the SVR aims to approximate a continuous-valued function. This approximation is achieved by minimizing model complexity while simultaneously constraining prediction errors within a user-defined margin [39,40]. Data points that lie outside this error margin, designated as support vectors, are critical in defining the regression function.

All algorithm scripts were written in PyCharm Community Edition (free) environment, using *scikit-learn*, a widely used open-source machine learning library for Python [41].

Each ML algorithm’s hyperparameters were fine-tuned by the method or technique called BayesSearchCV in the *Scikit-optimize* package [42] in PyCharm (PyCharm 2025), used in several ML publications [43,44,45]. This method leverages a probabilistic surrogate model to iteratively select the most promising hyperparameters based on prior evaluations [46]. Following Kavzoglu and Teke [47], we carried out a rigorous examination of the literature [48,49,50,51] to identify the most important hyperparameters and value range for each ML algorithm. BayesSearchCV incorporates cross-validation (CV), in k-folds, to ensure a robust evaluation of model performance across multiple data splits. In the present study, we set a 5-fold CV, in accordance with other publications [44,52].

### 3.3. Algorithm Performance Evaluation

The trained machine learning algorithms are evaluated on the testing dataset using three metrics. The first one is the coefficient of determination (*R*^2^), which expresses a measure of how well the predictions approximate the numerical simulation results. It ranges from 1, meaning a perfect fit, to negative values, which typically suggests that the model’s predictions are very inaccurate. The coefficient is determined as follows:

(18) $$R^{2}=1-\frac{\sum_{i=1}^{n}{\left(y_{t}-y_{p}\right)}^{2}}{\sum_{i=1}^{n}{\left(y_{t}-{\bar{y}}_{t}\right)}^{2}},$$

where *n* is the total number of samples in the dataset, $y_{t}$ is the true value of the output, $y_{p}$ is the predicted value of the output, and ${\bar{y}}_{t}$ is the mean value of the true output. The second metric we use is the root mean squared error (RMSE), given by the following:

(19) $$RMSE=\sqrt{\frac{1}{n}\sum_{i=1}^{n}{\left(y_{t}-y_{p}\right)}^{2}},$$

A lower RMSE is an indication of better model performance. The third performance metric we use is the mean absolute error (MAE), calculated from the following equation:

(20) $$MAE=\frac{1}{n}\sum_{i=1}^{n}\left|y_{t}-y_{p}\right|,$$

Obviously, a lower MAE indicates a better performance of the ML algorithm.

### 3.4. Interpretability of the Predictions

Machine learning models, such as the ones employed in the present study, are frequently characterized as a black box, meaning that the algorithm’s internal structure (i.e., how it makes predictions) is too complicated for any human to understand [53,54]. Understanding why certain decisions or predictions have been made makes the ML model transparent.

The most frequently used tool to interpret ML predictions is to use the theoretical concept of Shapley values, which originates from cooperative game theory introduced by Shapley [55]. Štrumbelj and Kononenko [56], in their study, illustrated how Shapley values can be used to determine the contribution of each feature in an ML model. In general, computing these values exactly is hard and computationally expensive [57]. The Shapley values method was later popularized by Lundberg and Lee [58], introducing the so-called Shapley Additive exPlanations (SHAP) values and including several SHAP variants (e.g., KernelSHAP and TreeSHAP) for specific ML models. For example, TreeSHAP is designed specifically for tree-based algorithms such as Decision Trees and XGBoost. In the present study, we used the SHAP values analysis on the ML algorithm that simultaneously had the highest $R^{2}$ values and lower error values.

## 4. Conclusions

The present study demonstrated the potential of three machine learning algorithms, Random Forests (RF), XGBoost, and Support Vector Regression (SVR), for providing valuable insights and predictive capabilities for the flat die extrusion of polymers for the production of sheets and films. These three algorithms were trained and tested on a computationally generated dataset encompassing a wide range of processing parameters and material properties. Two of the algorithms (XGBoost and SVR) exhibited a high degree of accuracy in predicting critical output targets such as thickness variation, pressure drop, and the lowest wall shear rate. The superior performance of XGBoost was evidenced by higher $R^{2}$ values and lower error metrics compared to Support Vector Regression (SVR). The application of SHAP analysis provided a crucial layer of interpretability, elucidating the importance of various input features and their impacts on the predicted outcomes (targets). The analysis highlighted the dominant influence of the polymer shear-thinning exponent and, to a lesser extent, the impact of the gap beneath the restrictor and the throughput rate on the transverse thickness variation. Other input parameters had a much smaller influence. Pressure drop was mainly influenced by the polymer consistency index, the throughput rate, the shear-thinning exponent, the consistency dependence on temperature, the temperature of the incoming polymer melt, and its thermal conductivity, in that order of importance. For the lowest wall shear rate, the throughput rate had, by far, the greatest influence, followed by the gap beneath the restrictor on the plane of symmetry and the consistency index.

## Figures and Tables

**Table 1:** Comparison of the predictive performance for the testing dataset of the three ML algorithms used.

Output (Target)	Random Forest (RF)			XGboost			Support Vector Regression (SVR)		
	$R^{2}$	RMSE	MAE	$R^{2}$	RMSE	MAE	$R^{2}$	RMSE	MAE
Thickness variation, h	0.807	1.261	0.914	0.954	0.591	0.452	0.924	0.821	0.672
Pressure drop, Δp	0.812	2.909	1.897	0.961	1.218	0.895	0.973	1.119	0.797
Lowest wall shear rate, ${\dot{\gamma }}_{w,min}$	0.955	6.255	4.631	0.985	1.619	0.927	0.977	2.238	1.238

**Table 2:** Polymer viscosity (measured at 225 °C) and temperature sensitivity in the study by Vlcek et al. [26].

Polymer	Power-Law Viscosity Model	Temperature Sensitivity, b (°C^−1^)
LLDPE-A	$\eta =3162{\dot{\gamma }}^{-0.3}$	0.022
LLDPE-B	$\eta =7914{\dot{\gamma }}^{-0.46}$	0.019
HDPE	$\eta =28,840{\dot{\gamma }}^{-0.68}$	0.02

**Table 3:** Inputs, outputs, and notation of the features and targets and corresponding value range.

Type	Notation	Description	Min–Max Value Range
Input	H1 (mm)	-	0.66–1.00
Input	H2 (mm)	-	0.66–1.00
Input	H3 (mm)	-	0.66–1.00
Input	H4 (mm)	-	0.68–1.00
Input	H5 (mm)	Bar restrictor	0.68–1.00
Input	H6 (mm)	locations	0.71–1.00
Input	H7 (mm)	-	0.73–1.00
Input	H8 (mm)	-	0.76–1.00
Input	H9 (mm)	-	0.84–1.00
Input	H10 (mm)	-	0.87–1.00
Input	H11 (mm)	-	0.87–1.00
Input	Q (kg/h)	Mass flow rate	100–2000
Input	$m_{ref}$ (Pa·s^n^)	Consistency index	5000–35,000
Input	n	Power-law index	0.25–0.55
Input	b (°C^−1^)	Temperature sensitivity	0.015–0.05
Input	ρ (kg/m^3^)	Melt density	750–1000
Input	$C_{p}$ (J/kg·K)	Specific heat capacity	1600–2300
Input	k (W/m·K)	Thermal conductivity	0.15–0.25
Input	$T_{in}$ (°C)	Melt entering temperature	190–230
Input	$T_{die}$ (°C)	Die walls temperature	190–230
Output	$h_{v}$ (%)	Thickness variation	0.635–11.20
Output	Δp (MPa)	Pressure drop	5–30.35
Output	${\dot{\gamma }}_{w,min}$ (s^−1^)	Lowest wall shear rate	5.763–130.41

## Data Availability

The original contributions presented in this study are included in the article. Further inquiries can be directed to the corresponding author(s).

## Abbreviations

The following abbreviations are used in this manuscript:

BOPP	Biaxially oriented polypropylene
CV	Cross validation
GNF	Generalized Newtonian fluid
HDPE	High-density polyethylene
LDPE	Low-density polyethylene
LLDPE	Linear low-density polyethylene
MAE	Mean absolute error
ML	Machine learning
PFAS	Per- and poly-fluorinated substances
PPAs	Polymer processing aids
RF	Random forest
RMSE	Root mean squared error
SHAP	Shapley additive explanations
SVR	Support vector regression
XGBoost	Extreme gradient boosting
